# Sex modifies the relationship between age and neurovascular coupling in healthy adults

**DOI:** 10.1177/0271678X231167753

**Published:** 2023-04-05

**Authors:** Jodie L Koep, Bert Bond, Alan R Barker, Stefanie L Ruediger, Faith K Pizzey, Jeff S Coombes, Tom G Bailey

**Affiliations:** 1 Physiology and Ultrasound Laboratory in Science and Exercise, Centre for Research on Exercise, Physical Activity and Health, School of Human Movement and Nutrition Sciences, The University of Queensland, Brisbane, Australia; 2 Children's Health and Exercise Research Centre, Sport and Health Sciences, College of Life and Environmental Sciences, University of Exeter, Exeter, UK; 3 School of Nursing, Midwifery and Social Work, The University of Queensland, Brisbane, Australia

**Keywords:** Ageing, cerebral blood flow, hyperaemia, posterior circulation, transcranial Doppler

## Abstract

Neurovascular coupling (NVC) is the matching between local neuronal activity and regional cerebral blood flow (CBF), but little is known about the effects of age and sex on NVC. This study aimed to investigate the relationships and interaction between age and sex on NVC. Sixty-four healthy adults (18–85 years, N = 34 female) completed a visual stimulus evoked NVC assessment to a flashing checkerboard. NVC responses were measured in the posterior cerebral artery (PCAv) using transcranial Doppler ultrasound. A hierarchical multiple regression was used to determine the relationships between age, sex, and the age by sex interaction on NVC. There was a significant age by sex interaction for baseline (P = 0.001) and peak PCAv (P = 0.01), with a negative relationship with age in females (P < 0.005), and no relationship in males (P ≥ 0.17). NVC responses as a percent increase from baseline showed a significant age by sex interaction (P = 0.014), with a positive relationship with age in females (P = 0.04) and no relationship in males (P = 0.17), even after adjusting for baseline PCAv. These data highlight important sex differences, with an association between age and NVC only apparent in females but not males, and thus a need to account for sex dependent effects of ageing when investigating cerebrovascular regulation.

## Introduction

Adequate regulation of cerebral blood flow (CBF) is vital given the high metabolic requirements of the brain, paired with its limited substrate storage capacity.^1^ In cases where CBF fails to match the local and regional energy demands of the brain, this can lead to chronic brain injury and is often associated with cognitive impairment.^2^ The response of adjusting local CBF to meet increased energetic demands of activated neurons by functional hyperaemia is termed neurovascular coupling (NVC). With advancing age in healthy individuals, declines in cognition and increased risk of cerebrovascular diseases are observed.^3,4^ Experimental animal models have shown a causal link between impairments in NVC and cognitive decline.^5^ In humans, a decline in NVC responses is shown in subjects with mild cognitive impairment, and is predictive of future dementia risk.^6^ In older adults there is a reduction in global and regional CBF,^7^ and these are associated with an increased risk of age associated neurodegenerative disease.^8^ Whether these declines translate to impairments in the regulation of CBF with advancing age and ability to match the requirements of activated brain regions via NVC is poorly understood.^9^

There is evidence to suggest reductions in NVC are observed in healthy ageing,^10–13^ however, this is not a universal finding,^14–19^ and the most consistent evidence only exists in animal models.^20–22^ At present there is no evidence on how the relationship between NVC and age changes through the entire adult lifespan, inclusive of middle-aged groups where vascular remodelling and neuronal loss occur.^23–26^ An important consideration yet to be addressed is the effects of sex on NVC responses, and whether any relationships between age and NVC may be sex dependent. This is likely, given the effects of ageing on the vasculature, and many underpinning mechanisms of NVC may be largely sex specific.^27,28^ In particular, it will be important to determine whether the menopausal transition, characterised by declines in female sex hormones and endothelial dysfunction^29^ are associated with declines in NVC. While a recent study reported no age or sex effect on NVC^19^ some limitations are worth noting. Importantly, Leacy et al.^19^ did not investigate the interaction of age and sex to determine if the effects of sex are dependent on age. This is important given the protective effects of estrogen on the vasculature, and gradual declines in estrogen over the.^30^ Additionally, only 2 individuals in the study by Leacy et al. were over the age of 60 years, thus limiting the conclusions regarding the effects of older age (65+ years) on NVC. This is of particular importance given that the age associated increases in neurodegenerative diseases occur after 65 years of age.^4,31^

Prior research has showed dynamic kinetic-based changes to CBF during a cerebrovascular challenge (carbon dioxide breathing) to be more sensitive compared to amplitude based measures to differences with advancing age.^32,33^ Therefore, the inclusion of kinetic outcomes in addition to traditional amplitude-based inferences in the current study may offer additional insights into regulatory responses.

The purpose of the present study was to determine the relationship between age and sex on NVC responses of the posterior cerebral artery across the complete adult lifespan (18 to 85 years old). Additionally, we aimed to identify if any alterations with age are sex dependent, and the impact of female menopausal status on NVC responses. It was hypothesised that 1) age and NVC would display a negative relationship, indicative of a blunted NVC with advancing age; and 2) females would have a greater decline in NVC with advancing age, coinciding with the menopausal transition.

## Materials and methods

### Study design

Following baseline screening, participants completed one visit to the laboratory in a cross-sectional study design. Each participant underwent an assessment of neurovascular coupling via activation of the visual cortex using a flashing checkerboard task. Cerebrovascular and cardiovascular measures were recorded throughout the protocol.

### Ethical approval

All experimental procedures and protocols were approved by the University of Queensland ethics committee (2019001863), and the study conformed to the standards set by the Declaration of Helsinki. Written informed consent was obtained prior to participation in the study.

### Participants

Seventy-three participants volunteered to take part in this study. Nine were excluded due to poor PCAv signal quality. The final sample included sixty-four adults (including thirty-four females). Participants were recruited into age groups for young (18–35 years), middle aged (36–64 years) or older aged (65–85 years) adults, as done previously.^33^ Exclusion criteria included diagnosed arterial hypertension, smoking, any known cardiometabolic or respiratory disease, the use of any prescribed medications known to influence cardiovascular function (e.g., statins, thyroid medication), a body mass index (BMI) >35 kg/m^2^ and a resting systolic blood pressure (SBP) >139 mmHg or diastolic blood pressure (DBP) 89 mmHg. Participants were recruited based on biological sex assigned at birth. Naturally menstruating pre- menopausal females (N = 8) were tested in the follicular phase *(days 1–14)* to control for hormones and allow comparisons between sexes.^34^ Pre-menopausal females on the combined contraceptive pill (N = 10) were tested during the inactive pill phase (*days 1–7*). Any pre-menopausal females with an irregular menstrual cycle or those using the progesterone only contraceptive pill were excluded. Post-menopausal females on hormone replacement therapy were also excluded. Included female participants self-reported menopausal status via questionnaire and were categorised into either premenopausal (regular periods), perimenopausal, early post-menopausal (1–3 years following last menstrual period) or late post-menopausal (6+ years following last menstrual period).^35^

### Experimental design

Participants were required to fast for a minimum of three hours, and refrain from nitrate rich foods for 12 hours prior to testing. In addition, participants were required to abstain from vigorous physical activity, caffeine and alcohol consumption for 24 hours prior to testing, which was ascertained via questionnaire upon arrival. Body mass and stature were measured according to standard procedures to the nearest 0.1 kg and 0.1 cm, respectively. Physical activity levels were assessed via the Active Australia survey,^36^ and reported as metabolic equivalent (MET) minutes per week.^37^ This survey has been validated against accelerometry data in healthy middle-aged adults,^38^ and agrees with other self-reported physical activity surveys.^39^ Following completion of the survey, participants were required to rest in a darkened, temperature-controlled laboratory (∼23°C) for 15 minutes in the supine position prior to commencement of the NVC protocol, with continual measurement of all cardiorespiratory and cerebrovascular variables.

### Blood pressure and cardiorespiratory measures

During the protocol, beat-by-beat blood pressure was measured continuously by finger volume-clamp method (Finapres medical systems, NOVA, Netherlands). Participants wore a face mask (Hans Rudolph Inc, Shawne, USA) with gas and flow analysis to measure end-tidal carbon dioxide concentrations (P_ET_CO_2_) using a gas analyser (ADInstruments, ML206, Colorado Springs, USA), which was calibrated prior to each participant via known concentrations of oxygen (4.01%) and carbon dioxide (16.10%). Heart rate was assessed using a three-lead echocardiogram (ECG) (Finapres medical systems, NOVA, Netherlands). All data were sampled continuously (Powerlab; model – 8/30, ADInstruments) and stored at 200 Hz using an analogue-to-digital converter interfaced with a laptop computer (Lab Chart version 8, ADInstruments) for offline analysis.

### Cerebrovascular measures

A 2-MHz transcranial Doppler ultrasound probe (Spencer Technologies, ST3, Redmond, WA) was used to assess cerebral blood velocity as an index of CBF of the left posterior cerebral artery (PCAv).^40^ Insonation of the PCA was performed through the trans-temporal window using previously described guidelines.^41^ The Doppler signal was acquired optimised and secured using an adjustable headset for unilateral PCAv assessment (adult M600 bilateral head frame; Spencer Technologies). Beat-by-beat PCAv was sampled continuously (Lab Chart version 8, ADInstruments).

### Neurovascular coupling

The NVC test evoked changes in PCAv in response to activation of the visual cortex. This consisted of five cycles of repeated, alternating, 30 seconds eyes closed followed by 30 seconds eyes open whilst focusing on a flashing checkerboard. This protocol was completed according to standardised guidelines, shown to be sensitive to neurodegenerative disease.^42^ The PCAv response to five cycles was exported on a breath-by-breath and beat-by-beat basis and used for data analysis.^42^ The time aligned NVC analysis included beat-to-beat (cardiovascular and cerebrovascular) and breath-by-breath (respiratory) data which were cubic spline interpolated at 5 Hz using a custom-built MATLAB code (The MathWorks, Natick, MA, USA).^42^ Data were averaged over the five transitions to provide a single response for each participant^43^ (**Supplementary figure 1**), except in cases where one transition was characterised by additional noise in the signal and then four cycles were used (N = 13). Outcomes of interest from this custom-built software included baseline PCAv (15 second average prior to eyes open stimulus), absolute peak PCAv (1 second average) during the 30 seconds of task engagement, relative percent increase between eyes closed baseline and peak PCAv during task engagement (equation (1)), as well as time to peak PCAv from stimulus onset, cerebrovascular conductance index (CVC) and cerebrovascular resistance index (CVRi) at baseline and peak (equations (2) and (3) respectively). Incremental area under the curve (AUC) during the 30 seconds following stimulus onset was calculated using the trapezoid rule as an average across the five transitions adjusted for baseline PCAv (GraphPad, Prism, version 9). Instrumentation and completion of the protocol was conducted by the same researchers in all participants and confirming that the participants’ eyes were closed and open during the respective trials, and that cerebrovascular (PCAv), cardiovascular (blood-pressure) and respiratory (P_ET_CO_2_) data returned to resting levels between cycles.

(1) relative change (%)=((peak PCAv−Baseline PCAv)/Baseline PCAv) * 100

(2) $${\mathrm{PCA}}_{\mathrm{CVC}}=\mathrm{PCAv}\;(cm/s)/\mathrm{mean}\;\mathrm{arterial}\;\mathrm{pressure}\;(\mathrm{MAP})\;(\mathrm{mmHg})$$

(3) $${\mathrm{PCAC}}_{\mathrm{VRi}}=\mathrm{MAP}\;(\mathrm{mmHg})/\mathrm{PCAv}\;(cm/s)$$

### Kinetic NVC response

Data were averaged across the five transitions and baseline-corrected for the 15 seconds preceding the onset of the visual stimulus and analysed using a mono-exponential model with time delay using GraphPad Prism (GraphPad Software, San Diego, CA) as follows: PCAv(t) = ΔPCAvA (1 − e −(t-TD/τ)), where PCAv(t) is the PCAv at a given time (t), ΔPCAvA is the amplitude change of PCAv from baseline to its asymptote, TD is the time delay and τ is the time constant (time taken to reach 63% of the response amplitude and it reflects the rate of increase in PCAv), in accordance with kinetic modelling in previous work.^32,44,45^ Mean response time (MRT) was calculated as the sum of the model derived τ and the TD. The model was fitted from the start of the exponential rise until a deviation from a visual steady state was observed. All models were then checked by two independent researchers for consistency, and any disagreements discussed until a consensus was reached. Acceptability of appropriate fit was determined as; goodness of fit R^2 ^>^ ^0.50, and normality of residuals. The precision of the derived τ was quantified using 95% confidence intervals.

### Statistical analyses

Statistical analyses were conducted using SPSS (version 25; IBM, Armonk, New York). All data were normally distributed as assessed by visual inspection of Q-Q plots and homoscedasticity of the studentized residuals plotted against the predicted values. All data are presented as mean ± SD. Differences in participant characteristics were explored using a two-way analysis of variance (ANOVA) with sex (male, female) and age (young, middle, older) as the independent variables. Post-hoc comparisons using a Bonferroni correction were performed to identify pairwise differences. Repeated measures ANOVA was used to determine the increase evoked by the visual stimulus for PCAv and PCAv CVC, with effect sizes displayed as partial eta squared (ηp^2^) and interpreted as <0.06 = small, 0.06–0.14 = moderate and >0.14 = large.^46^ For aim 1) a hierarchical multiple regression was used to determine the relationships between age (years) (model 1) and sex (model 2) with all baseline and NVC variables of interest. For aim 2) an interaction term of age × sex (model 3) was added to address whether sex moderated the effects of age by assessing the differences in regression slope coefficients between males and females on all variables of interest. The outputs for model 1 and 2 included the slope coefficient (unstandardised β), the explained variance of the full model (R^2^) and the significance of the relationship (P value). For model 3 the output included slope coefficients (unstandardised β) of the interaction terms for males by age, and for females by age. The P value describes whether there was a significant difference in slope coefficients between males and females with advancing age (significant interaction), and the R^2^ denotes the degree of explained variance of the entire regression model with the interaction term included. In order to adjust for any variance explained by body mass and physical activity levels; BMI (kg/m^2^) and self-reported physical activity (MET.mins per week) were added to the model. Lastly, MAP was added to the model to adjust for any variance on NVC outcomes explained by changes in perfusion pressure. For relative NVC (%) baseline PCAv was added to the model to determine if any variance was explained by changes in baseline PCAv.

A simple linear regression was run to investigate the influence of menopause on variables of interest in a female only model. The model investigated the relationship between early post-menopause (1–3 years LMP), and late post-menopause (6+ years LMP) compared to pre-menopausal females on variables of interest. In order to account for any variance explained by differences in age between menopausal groups, age was added to the model. For all regression analyses linearity was established by visual inspection of a scatterplot and there was no evidence of multicollinearity, as evidenced by no tolerance values less than 0.236. Although some data points were identified as above 2–3 standard deviations from the mean, none were deemed implausible and removed. Statistical significance was accepted at an alpha of P < 0.05.

## Results

Participants were recruited into young (N = 20, 10 female, age 26.7 ± 2.6, range = 22–32 years), middle aged (N = 26, 14 female, age 52.9 ± 7.7, range = 35–63 years) and older groups (N = 18, 10 female, age 70.2 ± 3.7, range = 65–77 years). Participant characteristics of the cohort can be seen in Table 1, highlighting the effects of age and sex on indicated variables. Significant age by sex interactions were present for height (P = 0.009), weight (P = 0.006), BMI (P = 0.01), PCAv (P = 0.003) and CVC respectively (P = 0.006). Pairwise comparisons for baseline PCAv and CVC show significant differences between males and females in young adults (P = 0.001 and P = 0.002 respectively). Additionally, in females, there were differences present between all age groups for PCAv and CVC (P < 0.02). For variables of BMI and weight, there were significant differences present in older adults, with a greater BMI (P = 0.006) and weight (P = 0.002) in older males compared to females.

**Table 1.** Participant characteristics.

Variables	Total		Young (18–35 years)		Middle aged (35–64 years)		Older (65–80 years)	
	Males (N = 30)	Females (N = 34)	Males (N = 10)	Females (N = 10)	Males (N = 12)	Females (N = 14)	Males (N = 8)	Females (N = 10)
Age (years)	49.3 ± 18.6	49.5 ± 16.4	26.2 ± 2.8^a,b^	27.4 ± 2.3^a,b^	50.6 ± 8.7^a,c^	54.4 ± 6.7^a,c^	71.1 ± 3.5^b,c^	69.2 ± 3.9^b,c^
Height (cm)	174.0 ± 9.2^†^	171.8 ± 8.1	175.1 ± 6.9	169.6 ± 3.7	171.2 ± 10.5	175.7 ± 6.9	176.8 ± 9.7	165.5 ± 6.9
Weight (kg)	75.0 ± 13.2*^†^	73.3 ± 14.5	70.1 ± 11.0	71.7 ± 13.3	72.6 ± 10.0	78.3 ± 15.8	84.9 ± 16.0	63.5 ± 5.9
BMI (kg m^2^)	21.5 ± 3.0^†^	21.3 ± 3.6	20.0 ± 2.6	21.1 ± 3.7	21.1 ± 2.1	22.2 ± 3.7	23.9 ± 3.5	19.2 ± 2.3
PA (METmins · week^−1^)	1954 ± 1133	2147 ± 1246	1171 ± 352	2280 ± 1223	1927 ± 1059	2229 ± 1297	2223 ± 1806	1756 ± 1260
PCAv (cm/s)	40.1 ± 9.0^†^	42.9 ± 11.2	38.4 ± 8.9^b^	52.3 ± 11.6^b^	42.2 ± 9.2^c^	41.8 ± 8.1^c^	39.0 ± 9.3^b,c^	32.2 ± 5.0^b,c^
CVRi (mmHg/cms^−1^)	2.6 ± 0.6	2.5 ± 0.8	2.5 ± 0.5^a,b^	1.9 ± 0.7^a,b^	2.4 ± 0.5^a,c^	2.6 ± 0.7^a,c^	2.9 ± 0.8^b,c^	3.3 ± 0.5^b,c^
CVC (cms^−1^/mmHg)	0.4 ± 0.1^†^	0.4 ± 0.2	0.4 ± 0.1^a,b^	0.6 ± 0.2^a,b^	0.4 ± 0.1^a,c^	0.4 ± 0.1^a,c^	0.4 ± 0.1^b,c^	0.3 ± 0.05^b,c^
MAP (mmHg)	98.4 ± 11.9	100.2 ± 12.8	90.7 ± 7.0^a,b^	90.9 ± 9.4^a,b^	98.4 ± 10.3^a^	104.0 ± 13.2^a^	108.0 ± 12.9^b^	104.5 ± 9.7^b^
P_ET_CO_2_ (mmHg)	41.4 ± 6.2	40.2 ± 4.8	43.6 ± 5.4	39.0 ± 3.6	42.2 ± 4.5	41.1 ± 5.1	37.3 ± 7.7	39.5 ± 5.6
HR (bmp)	61.5 ± 9.1	63.0 ± 6.6	59.7 ± 8.9	60.9 ± 7.7	64.0 ± 10.6	63.4 ± 6.8	60.1 ± 6.5	64.9 ± 4.3

Data presented as mean ± SD. Data were compared using a two-way ANOVA with main effects of age (young, middle, older) and sex (male, female). Asterix * indicates significant difference between males and females (P < 0.05). When main effect of age is present post-hoc pairwise comparisons (Bonferroni) reveal where significant differences lie: ^a^young vs middle, ^b^young vs older, ^c^middle vs older. Symbol † indicates significant age by sex interaction effect (P < 0.05). BMI: body mass index; PA: physical activity; PCAv: posterior cerebral artery; CVRi: cerebrovascular resistance index; CVC: cerebrovascular conductance index; MAP: mean arterial pressure; P_ET_CO_2_: end-tidal carbon dioxide; HR: heart rate.

### Baseline cardiovascular and cerebrovascular data

The relationship between age and sex and baseline cardiovascular and cerebrovascular data are shown in Table 2. These data are presented for age (model 1), age and sex (model 2) and the age by sex interaction (model 3).

**Table 2.** Baseline cardiovascular and cerebrovascular data.

Baseline variables	Model 1: Age			Model 2: Age and Sex (M = 30 F = 34)					Model 3: Interaction (Age × Sex)			
				Age		Sex		
	P-value	R^2^	β	P-value	β	P-value	β	R^2^	P-value	R^2^	β Males	β Females
PCAv (cm/s)	**0.013**	0.096	−0.19 ± 0.07	**0.011**	−0.19 ± 0.07	0.20	3.15 ± 2.45	0.12	**0.001**	0.26	0.02 ± 0.09	−0.42 ± 0.10
CVRi (mmHg/cms^−1^)	**<0.001**	0.24	0.021 ± 0.005	**<0.001**	0.021 ± 0.005	0.68	0.067 ± 0.16	0.25	**0.019**	0.31	0.011 ± 0.006	0.032 ± 0.007
CVC (cms^−1^/mmHg)	**<0.001**	0.22	−0.004 ± 0.001	**<0.001**	−0.004 ± 0.001	0.23	0.036 ± 0.029	0.24	**0.002**	0.36	−0.001 ± 0.001	−0.006 ± 0.001
MAP (mmHg)	**<0.001**	0.32	0.40 ± 0.07	**<0.001**	0.40 ± 0.08	0.64	−1.2 ± 2.6	0.33	0.93	0.33	0.40 ± 0.10	0.41 ± 0.11
P_ET_CO_2_ (mmHg)	0.37	0.013	−0.036 ± 0.04	0.39	−0.034 ± 0.04	0.41	−1.1 ± 1.4	0.024	**0.03**	0.098	−0.11 ± 0.05	0.06 ± 0.06
Heart Rate (bpm)	0.52	0.007	0.037 ± 0.057	0.54	0.035 ± 0.057	0.48	−1.4 ± 1.9	0.015	0.53	0.021	0.001 ± 0.079	0.074 ± 0.084

Data presented as mean ± SD. Bold indicated significant relationship (P < 0.05). Model 1 presents the relationship between age and the indicated variable. Model 2 presents the addition of sex to the model and the relationship between age and sex with the indicated variable. Model 3 indicates if sex moderates the relationship between age and the indicated variable, with individual beta-coefficients shown for males and females. In model 1,2 and 3 the R^2^ value reflects the full model. In model 1 and 3 β represents the unstandardised beta coefficient representing the change in variable units for every year increase in age. In model 2 the β coefficient provides the difference in females vs males in the variable units. PCAv: posterior cerebral artery velocity; CVRi: cerebrovascular resistance index; CVC: cerebrovascular conductance index; MAP: mean arterial pressure; P_ET_CO_2_: end-tidal carbon dioxide.

**Figure 1.**
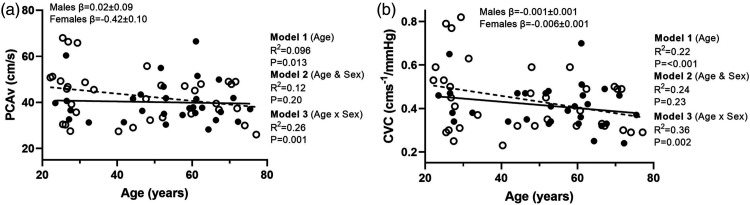
Linear regression analysis demonstrating the relationships between age and (a) baseline posterior cerebral artery and (b) baseline cerebrovascular conductance index. The solid line represents the regression fit for males (-•-) and the dotted line is the regression fit for female participants (-○-). Hierarchical regression models (P value and R^2^) are presented for the relationship with age (model 1), with sex (model 2) and the moderator relationship of the sex dependent relationship with age (model 3). Slope coefficients (β) of the relationships with age are presented for males and females separately (model 3).

There was a negative relationship between baseline PCAv and age (P = 0.013) (Figure 1(a)). With the addition of sex to the model there was no relationship between baseline PCAv and sex (P = 0.20). However, there was a significant age by sex interaction (P = 0.001), with a negative relationship with age in females (P < 0.001, β = −0.4 ± 0.10), which was not present in males (P = 0.83, β = 0.02 ± 0.09). With the addition of MAP, this did not explain any of the variance in baseline PCAv (P = 0.26, R^2^ = 0.02).

Figure 1(b) shows there was a negative relationship between baseline PCAv CVC and age (P < 0.001). With the addition of sex to the model there was no relationship (P = 0.23). There was a significant age by sex interaction for CVC (P = 0.002), with a negative relationship between CVC and age in females (P < 0.001, β = −0.006 ± 0.001), which was not present in males (P = 0.26, β = −0.001 ± 0.001).

The addition of physical activity levels and BMI did not significantly explain any variation for all baseline relationships (P > 0.3, R^2 ^<^ ^0.02) and so was not included in the full model.

### Neurovascular coupling response

**Figure 2.**
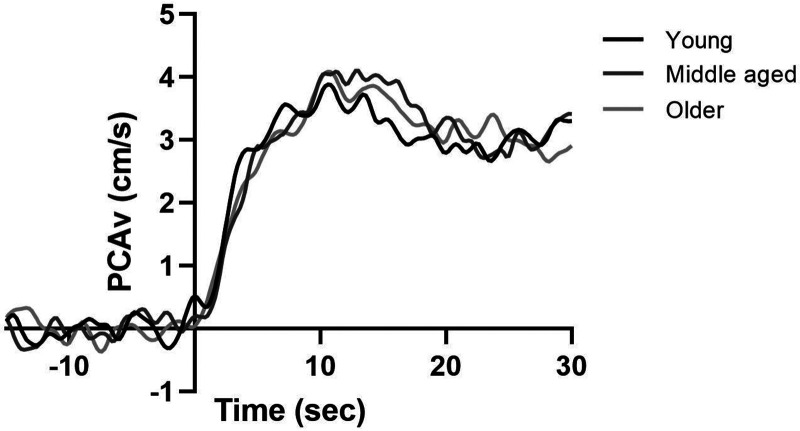
Baseline corrected PCAv responses during the 30 second visual stimulus, expressed as group responses in young, middle aged, and older adults.

Baseline corrected NVC responses in young, middle aged, and older adults across the 5 transitions can be seen in Figure 2. Across all participants the NVC test evoked an increase in PCA mean velocity and CVC from baseline to peak (PCAv: 41.6 ± 10.2 vs 47.2 ± 11.1 cm/s, P < 0.001, ηp^2^ = 0.75; PCAv CVC: 0.43 ± 0.13 vs 0.51 ± 0.15 cms^−1^/mmHg, P < 0.001, ηp^2^ = 0.77) indicating a sufficient visual stimulus of increasing the energetic demands was met by the NVC test. The peak cardiovascular and cerebrovascular NVC data can be seen in Supplementary Table 1.

**Figure 3.**
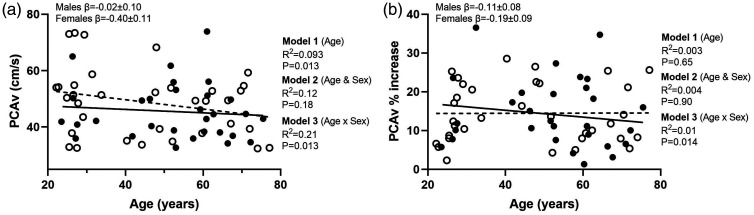
Linear regression analysis demonstrating the relationships between age and peak (a) posterior cerebral artery and (b) percent increase in PCAv. The solid line represents the regression fit for males (-•-) and the dotted line presents the regression fit for female participants (-○-). Hierarchical regression models (P value and R^2^) are presented for the relationship with age (model 1), the relationship with sex (model 2) and the moderator relationship of the sex dependent relationship with age (model 3). Slope coefficients (β) of the relationship with age are presented for males and females separately (model 3).

The relationship between age and sex and the cardiovascular and cerebrovascular responses to the visual stimulus are shown in Table 3. These data are presented for age (model 1), age and sex (model 2) and the age by sex interaction on the relationship between age and variables of interest (model 3). As also shown in Figure 3(a), peak PCAv responses during the visual stimulus showed a negative relationship with age (P = 0.014). With the addition of sex to the model this did not account for any additional variance in responses (P = 0.18). However, there was a significant age by sex interaction on peak PCAv responses (P = 0.013) with a negative relationship in females with age (P = 0.005, β = −0.40 ± 0.11), and no relationship in males (P = 0.82) (β = −0.023 ± 0.10).

**Table 3.** Peak cardiovascular and cerebrovascular responses to visual stimuli.

Peak variables	Model 1: Age			Model 2: Age and Sex (M = 30 F = 34)					Model 3: Interaction (Age × Sex)			
				Age		Sex						
	P-value	R^2^	β	P-value	β	P-value	β	R^2^	P-value	R^2^	β Males	β Females
PCAv (cm/s)	**0.013**	0.093	−0.20 ± 0.08	**0.012**	−0.20 ± 0.08	0.18	3.6 ± 2.6	0.12	**0.013**	0.21	−0.02 ± 0.10	−0.40 ± 0.11
Relative change (%)	0.65	0.003	0.028 ± 0.061	0.66	0.028 ± 0.061	0.90	0.28 ± 2.1	0.004	**0.014**	0.010	−0.11 ± 0.08	0.19 ± 0.09
PCAv AUC (cm/s/s)	0.61	0.004	−0.24 ± 0.47	0.59	−0.25 ± 0.47	0.66	7.3 ± 16.2	0.007	0.31	0.024	−0.69 ± 0.64	0.26 ± 0.69
Time to peak	0.13	0.037	0.084 ± 0.054	0.14	0.083 ± 0.055	0.79	0.51 ± 1.90	0.038	0.95	0.038	0.086 ± 0.076	0.080 ± 0.081
CVRi (mmHg/cms^−1^)	**<0.001**	0.24	0.021 ± 0.005	**<0.001**	0.021 ± 0.005	0.79	−0.044 ± 0.17	0.24	**0.03**	0.30	0.011 ± 0.006	0.032 ± 0.007
CVC (cms^−1^/mmHg)	**<0.001**	0.23	−0.004 ± 0.001	**<0.001**	−0.004 ± 0.001	0.31	0.034 ± 0.033	0.24	**0.014**	0.31	−0.002 ± 0.001	−0.007 ± 0.001
PCAv Amp (cm/s)	0.97	0.00	−0.001 ± 0.02	0.98	−0.001 ± 0.02	0.66	0.036 ± 0.82	0.005	0.18	0.051	−0.34 ± 0.03	0.03 ± 0.03
PCAv τ (s)	0.50	0.011	0.021 ± 0.031	0.50	0.021 ± 0.032	0.83	0.25 ± 1.1	0.013	0.63	0.019	0.004 ± 0.05	0.04 ± 0.04
PCAv TD (s)	0.59	0.009	−0.016 ± 0.03	0.46	−0.020 ± 0.03	**0.007**	2.6 ± 0.9	0.21	0.84	0.21	−0.016 ± 0.04	−0.026 ± 0.039
P_ET_CO_2_ (mmHg)	0.32	0.016	−0.04 ± 0.04	0.34	−0.04 ± 0.04	0.52	−0.88 ± 1.36	0.023	**0.025**	0.10	−0.12 ± 0.052	0.055 ± 0.056
Heart Rate (bpm)	0.81	0.001	0.021 ± 0.09	0.82	0.021 ± 0.09	0.92	0.30 ± 3.15	0.001	0.82	0.002	0.002 ± 0.13	0.043 ± 0.13

Data presented as mean ± SD. Bold indicated significant relationship (P < 0.05). Model 1 presents the relationship between age and the indicated variable. Model 2 presents the addition of sex to the model and the relationship between age and sex with the indicated variable. Model 3 indicates if sex moderates the relationship between age and the indicated variable, with individual beta-coefficients shown for males and females. In model 1, 2 and 3 the R^2^ value reflects the full model. In model 1 and 3 β represents the unstandardised beta coefficient representing the change in variable units for every year increase in age. In model 2 the β coefficient provides the difference in females vs males in the variable units. PCAv: posterior cerebral artery velocity; AUC: area under the curve; Amp: amplitude; τ: time constant; TD: time delay; CVRi: cerebrovascular resistance index; CVC: cerebrovascular conductance index; MAP: mean arterial pressure; P_ET_CO_2_: end-tidal carbon dioxide.

PCAv responses expressed as a relative percent increase revealed there was no relationship with age (P = 0.65) or sex (P = 0.90) (Figure 3(b)). However, sex moderated the effect of age (P = 0.014) showing a positive relationship in females with age (P = 0.035, β = 0.19 ± 0.09), and no relationship in males (P = 0.17, β = −0.11 ± 0.08). The addition of baseline PCAv to the model did not explain any of the variance in NVC responses (P = 0.17) or influence the relationships presented for model 1, 2 and 3. There was a negative relationship between peak PCAv CVC and age (P < 0.001). With the addition of sex to the model there was no relationship with peak PCAv CVC (P = 0.31). The relationship between peak CVC and age showed a significant age by sex interaction (P = 0.014), with a negative relationship between CVC and age in females (P < 0.001, β = −0.007 ± 0.001), which was not present in males (P = 0.13, β = −0.002 ±0.001).

PCAv area under the curve responses revealed there was no relationship with age (P = 0.61) or sex (P = 0.65) and no interaction effect of age and sex (P = 0.32). Time to peak PCAv responses showed no relationship with age (P = 0.13) or sex (P = 0.79), and no interaction effect of age and sex (P = 0.95).

Mono-exponential kinetic analyses are presented for 42 adults (N = 13 young, N = 18 middle aged, N = 11 older adults, 25 females), due to unacceptable model fit in 22 individuals. Reasons for data loss included poor model fit as identified by residuals (N = 6) and the inability to identify attainment of steady-state PCAv (N = 16). The PCAv response was well fitted by an exponential model (standard error of the τ: 0.67 ± 0.76). Kinetic responses for the τ revealed there was no relationship with age (P = 0.50) or sex (P = 0.83) and no interaction effect of sex (P = 0.63).

The addition of physical activity levels and BMI did not explain the variation in any of the NVC response variables (P > 0.13, R^2 ^<^ ^0.03) and so was removed from the full model. Additionally, peak MAP during the visual stimulus did not explain any of the variance in NVC responses (peak PCAv, relative change (%), PCAv AUC) (P > 0.38, R^2 ^<^ ^0.013), and so was not adjusted for.

### Effects of menopause on NVC

The relationships between NVC and menopausal status (pre, early-post, late-post) are shown in Table 4. In addition, the influence of age between menopausal groups has been accounted for. Baseline and peak PCAv and CVRi showed a significant relationship with menopausal status (P ≤ 0.02, R^2^ ≥ 0.15). This relationship was not explained by the change from pre-menopausal to post-menopausal groups (P > 0.71), but there was a significant relationship for baseline and peak PCAv and CVRi from pre-menopausal compared to late post-menopausal females (P < 0.005). The relationship between baseline and peak PCAv from pre-menopausal compared to late post-menopausal females was not contributed to by the inclusion of age to the model (P > 0.23). For baseline and peak CVRi there was a significant relationship with age (P < 0.03), however, the relationship with menopausal status persisted and was not accounted for by age (P < 0.004).

**Table 4.** Relationships between menopausal status and NVC.

NVC parameters	Model		Coefficients					
	P-value	R^2^	Early post-menopausal (57.0 ± 5.9 years, N = 7)		Late post-menopausal (66.6 ± 3.7 years, N = 13)		Age (49.5 ± 16.4 years, N = 34)	
			β	P	β	P	β	P
Baseline parameters								
PCAv (cm/s)	**0.02**	0.16	0.8 ± 4.7	0.87	−7.4 ± 3.6	**0.041**	−0.09 ± 0.09	0.27
MAP (mmHg)	**<0.001**	0.21	6.6 ± 4.9	0.19	5.0 ± 3.8	0.19	0.3 ± 0.1	**0.001**
CVRi (mmHg/cms^−1^)	**<0.001**	0.35	0.11 ± 0.29	0.71	0.69 ± 0.23	**0.004**	0.01 ± 0.01	**0.001**
CVC (cms^−1^/mmHg)	**0.001**	0.27	−0.02 ± 0.06	0.75	−0.09 ± 0.04	0.06	−0.003 ± 0.01	**0.02**
P_ET_CO_2_ (mmHg)	0.16	0.08	4.7 ± 2.6	0.17	3.1 ± 2.0	0.13	−0.08 ± 0.05	0.09
HR (bpm)	0.81	0.016	2.6 ± 3.9	0.51	1.5 ± 3.0	0.62	0.01 ± 0.07	0.85
Peak parameters								
PCAv (cm/s)	**0.02**	0.15	1.7 ± 5.1	0.74	−8.7 ± 3.9	**0.04**	−0.11 ± 0.09	0.23
MAP (mmHg)	0.20	0.07	9.8 ± 6.2	0.12	6.7 ± 4.8	0.17	0.01 ± 0.1	0.97
CVRi (cms^−1^/mmHg)	**<0.001**	0.34	0.09 ± 0.30	0.77	0.69 ± 0.24	**0.005**	0.01 ± 0.005	**0.03**
CVC	**0.002**	0.27	−0.02 ± 0.06	0.80	−0.09 ± 0.05	0.07	−0.01 ± 0.001	**0.01**
Relative PCAv (Δ%)	0.67	0.03	0.90 ± 4.1	0.83	3.7 ± 3.2	0.25	−0.02 ± 0.07	0.79
PCAv AUC (AU)	0.94	0.007	1.9 ± 31.7	0.95	9.5 ± 24.6	0.70	−0.36 ± 0.57	0.54
Time to peak (s)	0.23	0.07	−4.7 ± 3.7	0.20	0.69 ± 2.8	0.81	0.09 ± 0.07	0.20
HR (bpm)	0.85	0.01	−0.11 ± 6.1	0.99	3.9 ± 4.7	0.41	−0.03 ± 0.11	0.82
Kinetic parameters								
PCAv τ (s)	0.59	0.05	0.71 ± 2.7	0.79	−1.8 ± 1.6	0.28	0.05 ± 0.04	0.24
PCAv Amp Δ (cm/s)	0.22	0.11	1.8 ± 1.9	0.14	−1.3 ± 1.2	0.26	0.02 ± 0.03	0.57
PCAv TD (s)	0.14	0.16	1.8 ± 2.1	0.40	3.0 ± 1.3	**0.03**	−0.07 ± 0.04	0.07

Data presented as mean ± SD. Multiple linear regression investigating the influence of menopausal status on baseline and peak NVC responses. Model coefficients investigate the relationship between early post-menopause (1–3 years), and late post-menopause (6+ years) to a reference group of pre-menopausal women (34.0 ± 10.2 years) on the variables of interest. Age was added to the model to account for any variance explained by differences in age between groups. For variable of age, model coefficients represent the change in variable units for every year increase in age. Bold indicated significant relationship (P < 0.05). β-coefficients for early post-menopausal and late post-menopausal females are presented compared to reference group of premenopausal females. PCAv: posterior cerebral artery velocity; AUC: area under the curve; Amp: amplitude; τ: time constant; TD: time delay; CVRi: cerebrovascular resistance index; CVC: cerebrovascular conductance index; MAP: mean arterial pressure; P_ET_CO_2_: end-tidal carbon dioxide; HR: heart rate.

## Discussion

To our knowledge this is the first study to address the sex specific relationships of age on NVC across a wide age range (22 to 77 years), which is an important extension of previous work.^19^ The main findings were: 1) there was a negative relationship between age and peak PCAv and CVC responses to NVC; 2) this decline was sex specific and only apparent in females with advancing age, 3) the relative NVC response was preserved with advancing age, however, this was shown to be sex dependent with a positive relationship between age and NVC in females and no relationship with age in males, and 4) reduced peak PCAv and CVRi in females were only evident in late post-menopausal females (6+ years), compared to early post-menopausal and premenopausal females, and was not accounted for by age.

Declines in resting CBF and cerebral metabolic rate with advancing age have been previously reported.^47–49^ However, the majority of these studies only included measures of middle cerebral artery velocity (anterior circulation)^50–53^ or global CBF,^54^ and not the posterior circulation. This may be particularly important given the posterior circulation supplies the brainstem, which is critical for life functions and regulates respiratory and cardiac function.^55^ The declines in baseline PCAv with age in the current study are consistent with some,^56,57^ but not all studies.^10^ This discrepancy between the current study and Fluck et al.^10^ may be due to their data only including a small sample of older females (N = 8) and not accounting for sex differences. Our study found that in males, PCAv was preserved with age and decreases were only evident in the female sample, driving the overall negative relationship between PCAv and age. The current study found a ∼4 cm/s decrease in PCAv per decade in females, and when comparing late-postmenopausal females to pre-menopausal females this showed a ∼7.4 cm/s decrease in PCAv over the menopausal transition. A number of reasons could be responsible, but most notably, the loss of estrogen in post-menopausal females is proposed to result in greater declines in CBF in older females.^58,59^ The declines in PCAv were evident despite an increased MAP with advancing age, and hence greater driving force propelling blood through the vessels. Previous research has proposed that restricted blood flow in the posterior circulation may result in increases in blood pressure via the “selfish brain hypothesis”.^60–63^ This theory has been proposed in the etiology of hypertension, as well as a contributing factor to sex differences in CBF regulation with age. It has been hypothesised that greater increases in blood pressure in females may be resultant of, and protective against the declines in posterior blood flow to the brainstem.^63^ However, in the present study there was no relationship present between MAP and PCAv with age, with longitudinal data needed to explore the relationship between changes in MAP and PCAv over time.

This study is the first to document the kinetic responses of the PCAv during NVC and found no relationship with age. There was also no relationship between the relative NVC (%) with age. This corroborates previous findings,^15,16,18^ and most recently Leacy et al.^19^ who all found a preserved NVC in older compared to younger adults. Evidence, however, remains conflicting with other studies reporting increased,^64–66^ or reduced NVC responses with ageing.^67–70^ Importantly, our findings highlight the disparate NVC responses with age in males and females. In females a greater relative increase in PCAv during visual task engagement was observed with advancing age, which was in contrast to males, in whom NVC responses showed no relationship with age. This increased response with age in females occurred despite a comparable blood pressure response between males and females, and when accounted for statistically, the positive relationship between NVC responses and age in females was not explained by the increase in blood pressure with age. This highlights the need for future studies to account for the sex dependent effect of age when investigating cerebrovascular regulation and might cloud interpretations of ageing and cerebral blood flow studies where males and females are not investigated independently. The current findings build on recent work^20^ and highlight that when older adults (65+ years) and sex dependent relationships are considered, NVC does in fact change with advancing age, driven by older females. Future research whereby blood markers for estrogen and NO concentrations are sampled is required to further understand the effects of female sex hormones and menopause disentangled from the effects of age on the cerebrovascular circulation. Despite these differences with advancing age in females compared to males, it should be highlighted that this influence of age and sex only accounts for up to 21% of the variance, as reflected by the R^2^ values for peak PCAv and the NVC response respectively. This is comparable with peripheral vascular data, whereby the effects of age account for 16% of the flow mediated dilation response in females.^71^ This indicates the presence of other influencing factors, as well as between subject variability in the NVC response.

We hypothesised there would be a negative relationship between NVC responses and age, and to a greater extent in females than males. However, what is considered to be a healthy and optimal response with advancing age is largely unknown. Therefore, the greater NVC response with age in females compared to males observed here may be explained by a compensatory response of increased CBF to the posterior circulation, allowing older females to recruit additional neural circuits to maintain blood flow and energy requirements to the brain. We propose that this compensatory response may present impaired efficiency and a reduced capacity to redistribute CBF. Prior evidence using cognitive tasks has shown hyperactivated states to result in enhanced neurotoxicity, due to increased neural recruitment.^72^ Furthermore, studies using cognitive tasks provide support for elevated responses not representing a superior physiological response in the context of advancing age. This is supported by data from Beishon et al.^73^ who observed increased cerebrovascular velocity responses (∼44%) in the anterior circulation with advancing age to cognitive stimulation. This has also been documented in a review whereby Beishon et al.^6^ highlighted individuals with mild cognitive impairment displayed elevated CBF responses to NVC tasks compared to healthy controls. In order to delineate whether an elevated response is beneficial versus harmful in the long term, longitudinal studies are required to understand these age-related changes.

Contrasting findings on the relationship between age and NVC may be due to a number of methodological differences, including; study design, measurement techniques, sex, handedness of participants, number of trials, NVC task, nature of participants, and task engagement.^74^ Further, different haemodynamic markers characterising the NVC response may provide different results. This is evidenced in the current data, whereby the peak PCAv and percent increase in PCAv were associated with advancing age whereas the kinetic response and AUC showed no relationship with advancing age in both males and females. The lack of relationships in the time-based metrics may be unsurprising given that the NVC response occurs relatively instantaneously (within a few seconds),^75^ and the occurrence of this may only be compromised in neurovascular uncoupling and the onset of disease, though research is needed to confirm this. Further, time-based metrics are characterised by increased variability,^76^ which is also observed in the current data. In the present study, participants were characterised by high physical activity levels (2056 MET.mins per week), which has been suggested to protect against declines in cardiovascular^77^ and cerebrovascular function.^49,78^ When accounted for statistically, physical activity levels did not seem to have any relationship with NVC outcomes. However, this does not negate the potential for NVC responses reported to reflect the high average levels of the sample and thus a maintained ability to redistribute blood flow in response to visual stimulation and protect against vascular ageing. The effects of physical activity levels on NVC in ageing adults therefore needs to be specifically addressed using objective measures in future research.

Despite the novelties of the current study, there are a number of methodological considerations that should be considered in the interpretation of these findings. Firstly, the use of transcranial Doppler ultrasound as a surrogate measure of flow relies on the assumption that vessel diameter of the PCAv remains constant during visual stimulation.^75^ Recent evidence however, challenges this, suggesting that an increase in the diameter of the PCA is seen during visual stimulation.^79^ This would result in underestimation of the velocity based estimate of CBF changes under conditions of vessel dilation. It should also be noted that vascular remodelling of arteries^80^ and greater variability in cross sectional area may occur with age.^81^ Although this has not been addressed in the PCA, a smaller magnitude of diameter changes observed in older compared to young adults may result in a larger underestimation of flow in young adults and affect current conclusions on the effect of advancing age. Studies utilising MRI measures of the PCA to discern how age and sex effect diameter changes to an NVC task are required. A further limitation includes the use of a flashing checkerboard stimulus for the assessment of NVC. Given the lower task engagement of this metric to other approaches,^74^ differential NVC responses could be due to individual differences in task engagement, and a potential age effect of task engagement. Given that we only measured the left PCA, the possibility that age or sex influence the lateralisation of NVC responses also remains to be investigated, with the need for future research to include measures of right PCAv to account for this possibility. Further, the current study assessed pre-menopausal females in the follicular phase with differences in estrogen concentrations evident between early and late follicular phases.^82^ Thus, while efforts were made to control for cycle phase large inter- and intra-individual differences in estrogen concentration prevail even when controlling for cycle phase.^83^ In considering the interactions of sex we observed on the ageing response, future studies would benefit from larger sample sizes, given this variability. Most importantly, the cross-sectional nature of the current study limits the interpretations on the effects of age on NVC responses.

In conclusion, the present study shows a negative relationship between baseline and peak PCAv and PCA conductance index with advancing age, which was driven by females. This decrease in PCAv occurred in late post-menopausal females, with pre-menopausal and early post-menopausal females protected against declines. Additionally, we show a sex dependent NVC response, whereby NVC is preserved with age in males, and females are characterised by an increased NVC response with age. The underlying physiological reasons remain unknown, with further longitudinal evidence required to distinguish the effects of normal ageing on cerebrovascular responses to visual stimulation. Additionally, the current findings highlight the need for research to include direct measures of hormone concentrations to advance our understanding on the effects of sex hormones and menopausal transition on cerebrovascular regulation.

## Supplemental Material

sj-pdf-1-jcb-10.1177_0271678X231167753 - Supplemental material for Sex modifies the relationship between age and neurovascular coupling in healthy adults Click here for additional data file. Supplemental material, sj-pdf-1-jcb-10.1177_0271678X231167753 for Sex modifies the relationship between age and neurovascular coupling in healthy adults by Jodie L Koep, Bert Bond, Alan R Barker, Stefanie L Ruediger, Faith K Pizzey, Jeff S Coombes and Tom G Bailey in Journal of Cerebral Blood Flow & Metabolism
