# Supplementary material for: Sex modifies the relationship between age and neurovascular coupling in healthy adults
Source: J Cereb Blood Flow Metab. 2023 Apr 5;43(8):1254–66. doi: 10.1177/0271678X231167753 (PMC10369153; doi:10.1177/0271678X231167753)
Supplement: sj-pdf-1-jcb-10.1177_0271678X231167753 - Supplemental material for Sex modifies the relationship between age and neurovascular coupling in healthy adults [file sj-pdf-1-jcb-10.1177_0271678X231167753.pdf]

**Supplementary information:**

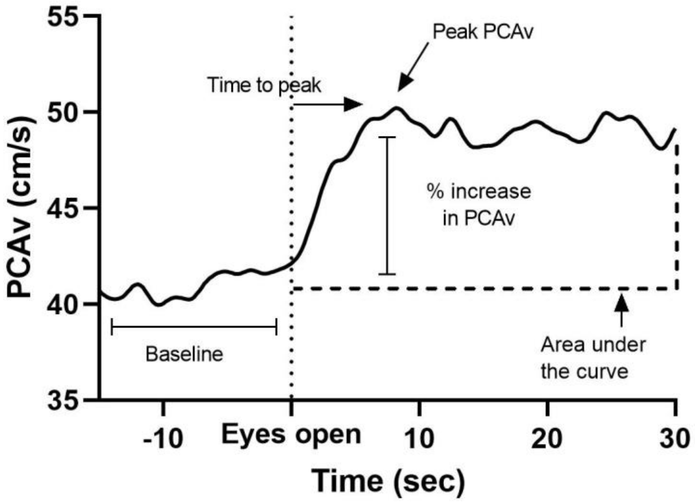


**Supplementary Figure 1.** Average representative trace of the posterior cerebral artery velocity averaged across 5 transitions during the visual stimulus, highlighting the outcomes derived from the neurovascular coupling assessment.

**Supplementary Table 1**. Peak cerebrovascular responses to visual stimulation

| **Variables** | **Total** | | **Young (18 - 35 years)** | | **Middle aged (35 - 65 years)** | | **Older (65 - 80 years)** | |
|---|---|---|---|---|---|---|---|---|
| | Males (N=30) | Females (N=34) | Males (N=10) | Females (N=10) | Males (N=12) | Females (N=14) | Males (N=8) | Females (N=10) |
| PCAv (cm/s) | 45.5±9.9† | 48.8±12.0 | 44.0±9.3[b] | 57.6±12.7[b] | 48.5±10.6[c] | 47.9±9.7[c] | 42.9±9.3[b,c] | 38.4±6.3[b,c] |
| CVRi (mmHg/cms$^{-1}$) | 2.6±0.6 | 2.6±0.8 | 2.5±0.5[b] | 2.0±0.8[b] | 2.5±0.6[c] | 2.7±0.7[c] | 3.0±0.8[b,c] | 3.4±0.5[b,c] |
| CVCi (cms$^{-1}$/mmHg) | 0.5±0.1† | 0.5±0.2 | 0.5±0.1[a,b] | 0.7±0.2[a,b] | 0.5±0.1[a,c] | 0.5±0.1[a,c] | 0.4±0.1[b,c] | 0.4±0.1[b,c] |
| NVC (%) | 14.1±9.2 | 14.5±7.6 | 15.4±9.3 | 10.3±5.4 | 15.2±8.4 | 14.9±8.1 | 11.0±10.9 | 19.3±6.8 |
| MAP (mmHg) | 97±10* | 109±13 | 96±10 | 105±15 | 97±11 | 120±11 | 99±10 | 113±14 |
| PCAv Amp (cm/s) | 6.3±2.5 | 6.0±2.6 | 6.4±2.4 | 4.8±1.9 | 7.2±1.6 | 6.8±3.1 | 5.1±3.3 | 6.0±2.3 |
| PCAv τ (s) | 4.6±2.3 | 4.3±4.2 | 4.4±2.1 | 2.6±2.1 | 4.5±1.5 | 5.8±4.9 | 4.8±3.5 | 3.9±1.6 |
| PCAv TD (s) | 0.2±1.2* | 2.7±3.3 | 0.08±1.1 | 3.5±1.3 | 0.4±1.4 | 3.3±4.4 | 0.2±1.0 | 1.0±0.6 |
| PCAv MRT (s) | 4.8±1.8 | 7.6±5.1 | 5.0±1.9 | 7.1±1.7 | 4.9±1.3 | 9.1±6.5 | 4.6±2.6 | 4.9±4.9 |

*Data presented as mean ± SD deviations. Data were compared using a two-way ANOVA with main effects of age (males, females) and sex (young, middle, older). Asterix * indicates significant difference between males and females (P<0.05). When main effect of age is present post-hoc pairwise comparisons (Bonferroni) reveal where significant differences lie: a=young vs middle, b=young vs older, c=middle vs older. Symbol † indicates significant age by sex interaction effect (P<0.05). Pairwise comparisons for the interaction effects for peak PCAv and CVC revealed a difference between males and females for young adults only (P=0.02 for both). In females there was a significant difference between for PCAv between all age groups (P≤0.04). For peak CVC, in females there was a difference between young adults compared to middle (P=0.001) and older aged adults (P<0.001). PCAv, posterior cerebral artery; CVRi, cerebrovascular resistance index; CVCi, cerebrovascular conductance index; NVC, neurovascular coupling; MAP, mean arterial pressure; Amp, amplitude; τ, time constant.*
